# Risk of lymphadenopathy from SARS-CoV-2 vaccination in Korea: a self-controlled case series analysis

**DOI:** 10.4178/epih.e2023090

**Published:** 2023-10-13

**Authors:** Mi-Sook Kim, Bongyoung Kim, Jeong Pil Choi, Nam-Kyong Choi, Jung Yeon Heo, Jun Yong Choi, Joongyub Lee, Sang Il Kim

**Affiliations:** 1 Medical Research Collaborating Center, Seoul National University Hospital, Seoul, Korea; 2 Department of Internal Medicine, Hanyang University College of Medicine, Seoul, Korea; 3 Department of Preventive Medicine, Seoul National University College of Medicine, Seoul, Korea; 4 Department of Health Convergence, Ewha Womans University, Seoul, Korea; 5 Department of Infectious Diseases, Ajou University School of Medicine, Suwon, Korea; 6 Department of Internal Medicine and AIDS Research Institute, Yonsei University College of Medicine, Seoul, Korea; 7 Department of Internal Medicine, College of Medicine, The Catholic University of Korea, Seoul, Korea

**Keywords:** COVID-19 vaccines, Lymphadenopathy, Self-controlled case series, Vaccine safety

## Abstract

**OBJECTIVES:**

To assess the risk of lymphadenopathy following severe acute respiratory syndrome coronavirus 2 (SARS-CoV-2) vaccination.

**METHODS:**

A self-controlled case series design was used to determine whether the risk of lymphadenopathy was higher in the 1-day to 42-day risk interval after coronavirus disease 2019 (COVID-19) vaccination compared to the control period. In addition, subgroup analyses were conducted according to baseline characteristics, time since vaccination, and sensitivity analyses adjusted for the length of the risk interval.

**RESULTS:**

The risk of developing lymphadenopathy in the risk interval (1-42 days) after COVID-19 vaccination compared to the control period was significantly increased, with a relative incidence (RI) of 1.17 (95% confidence interval [CI], 1.17 to 1.18) when the first, second, and third doses were combined. The RI was greater on the day of vaccination (1.47; 95% CI, 1.44 to 1.50). In subgroup analyses by baseline characteristics, a significantly increased risk or trend toward increased risk was observed in most subgroups except for those aged 70 years and older, with a significant increase in risk in younger individuals, those with a Charlson’s comorbidity index <5, and those who received mRNA vaccines (mRNA-1273>BNT162b2). Within the 1-day to 42-day post-dose risk period, the relative risk was highest during the 1-day to 7-day post-dose period (1.59; 95% CI, 1.57 to 1.60) compared to the control period, and then the risk declined. In the sensitivity analysis, we found that the longer the risk window, the smaller the RI.

**CONCLUSIONS:**

SARS-CoV-2 vaccination is associated with a statistically significant increase in the risk of lymphadenopathy, and this risk was observed only with mRNA vaccines.

## INTRODUCTION

A lymph node (LN) is a small gland that filters lymph, a fluid that circulates in the lymphatic system, and is the site of maturation of lymphocytes [1]. Lymphadenopathy, which refers to an abnormality in LN size and consistency, can occur due to various reasons, including bacterial, viral, or fungal infections, autoimmune disorders, and malignancies [2]. Reports have surfaced indicating the occurrence of lymphadenopathy after coronavirus disease 2019 (COVID-19) vaccination, which is thought to occur as the vaccine components enter the LNs through the lymphatic system and generate an immune reaction, causing proliferation of lymphocytes [3]. The S-specific TFH cell response in axillary LN fine-needle biopsies from 15 individuals in the clinical trial of the BNT162b2 vaccine could be interpreted as mechanistic evidence that lymphadenopathy can be induced as part of the normal immune response to vaccination [4]. Furthermore, the observation of S-binding germinal center B cells and plasmablasts in the draining LNs after a booster dose of the BNT162b2 vaccine provides the same rationale for lymphadenopathy due to the booster vaccination [5].

During the clinical trials of the mRNA-1273 vaccine, 11.6% of patients (5.0% placebo) experienced axillary swelling or tenderness after the first dose, and 16.0% (4.3% placebo) after the second dose. Considering a previous study reporting that 4.8% (4/83) of patients with influenza vaccination had axillary lymphadenopathy, COVID-19 vaccines may be associated with a higher incidence of lymphadenopathy than other vaccines [6]. Another study revealed that the pooled prevalence of axillary lymphadenopathy after COVID-19 vaccination was 37% (95% confidence interval [CI], 27 to 47) on positron emission tomography/computed tomography [7]. Furthermore, in a prospective observational study conducted in Jordan, the incidence of lymphadenopathy within 14 days of COVID-19 vaccination was 157.9 per 100,000, the highest of the 18 red-flag outcomes analyzed [8]. Hence, lymphadenopathy could be regarded as a common adverse event of COVID-19 vaccination.

While some studies have reported that there was no association between COVID-19 vaccination and the development of lymphadenopathy [9], a few studies have demonstrated an increase in lymphadenopathy caused by COVID-19 vaccines [10-12]. However, no previous studies have adopted the self-controlled case series (SCCS) design which was originally developed for the evaluation of vaccine safety [13]. Therefore, this study applied an SCCS design, aiming to determine whether there was an association between COVID-19 vaccination and the incidence of lymphadenopathy, and to clarify the clinical characteristics of lymphadenopathy that might occur after COVID-19 vaccination.

## MATERIALS AND METHODS

### Data source

This study was conducted using data from the National Health Insurance Service (NHIS) during the period from January 1, 2020 to December 31, 2021. The NHIS is a comprehensive national health insurance system that encompasses all citizens of the Korea and provides patient demographic, diagnostic, procedural, operative, and prescription data from all insurance claims, which are used for research purposes [14]. The Korea Disease Control and Prevention Agency (KDCA) used a linkage key of national identification numbers to connect its COVID-19 confirmed case and vaccination databases with the NHIS data. The immunization database linked by the KDCA contains information on the date of vaccination, the type of vaccine administered, and the dosage [15]. The COVID-19 confirmed case database includes the COVID-19 diagnosis date, death status, and date of death.

Upon integration of the NHIS and KDCA databases, unique study identification numbers were assigned to replace the national identification numbers. Subsequently, the data were rendered de-identified and access was restricted to authorized analysts only through an NHIS-controlled remote data analysis system.

### Inclusion/exclusion criteria of patients for the case-only study design

The SCCS design is a patient-only study design utilized to assess the causal contribution of COVID-19 vaccination to lymphadenopathy while controlling the bias from unmeasured time-invariant confounders [16]. Before applying the SCCS design, it is necessary to consider whether there is an event-related exposure or an event-related observation period [17]. Lymphadenopathy is usually temporary and exhibits mild symptoms that do not warrant discontinuation of vaccination. Moreover, deaths among patients with a claimed diagnosis of lymphadenopathy are frequently due to comorbidities that cause the lymphadenopathy, not the lymphadenopathy itself. Therefore, the standard SCCS approach was selected, assuming no relationship between death and lymphadenopathy, with follow-up censored at the time of death. The study included patients aged 18 years or older, regardless of COVID-19 vaccination status, who received medical care for lymphadenopathy between January 1, 2021 and December 31, 2021. Unvaccinated individuals were primarily included to account for seasonal effects in the analysis. The exclusion criteria were as follows: (1) patients with a history of lymphadenopathy in the previous 1 year; (2) patients with a presumptive diagnosis of the cause of lymphadenopathy within 3 months of the diagnosis of lymphadenopathy or within 12 months before the diagnosis, such as tuberculosis, plague, tularemia, chlamydial infection, human immunodeficiency virus, sporotrichosis, malignant neoplasm, hemangioma and lymphangioma, other neoplasms of lymphoid, hematopoietic tissue, sarcoidosis, and postmastectomy lymphedema syndrome; (3) patients with confirmed COVID-19; (4) patients whose COVID-19 vaccination records in the database of the KDCA were missing; (5) patients vaccinated through clinical trials; and (6) patients with 2 or more vaccination records within a single dose, or if the chronological order of vaccination series was reversed.

### Variables

The primary outcome variable was a healthcare visit due to lymphadenopathy, and the operational definition of lymphadenopathy in the NHIS database was determined by consensus of a panel of experts from the National Academy of Medicine of Korea as a claim with International Statistical Classification of Diseases and Related Health Problems 10th revision (ICD-10) codes: D36.0, I88.x, I89.x, L04.x, and R59.x. The study utilized the KDCA database to confirm the exposure to the COVID-19 vaccine, and it was confirmed that the ChAdOx1, BNT162b2, Ad26. COV2.S, and mRNA-1273 vaccines were administered during the study period. Using data from 2021, patients were categorized into age groups of 18-29 years, 30-39 years, 40-49 years, 50-59 years, 60-69 years, 70-79 years, and 80 years or older. Charlson’s comorbidity index (CCI) was also utilized to identify 19 comorbidities and weight them in order to divide patients into two groups: those with a score of 0-4 or 5 or more [18]. The comorbidities considered in the stratification analysis and those considered for exclusion were defined using ICD-10 codes and are presented in Supplementary Material 1.

### Statistical analysis

A summary of the baseline characteristics of the study population was presented by the timing of lymphadenopathy: those experiencing lymphadenopathy on the vaccination day, those developing it in the risk period (between days 1 and 42 after vaccination), and those experiencing it during the remaining period of the observation period of 365 days in 2021, which served as the control period. The length of the risk period was chosen to be 42 days because previous mRNA vaccine safety assessment studies have used a 42-day adverse event observation period [10], and because magnetic resonance imaging (MRI)-based axillary lymphadenopathy studies have shown that the prevalence of lymphadenopathy between 29-42 days after mRNA vaccination is 3 times higher than the baseline level [19]. Descriptive statistics were used to summarize categorical variables in terms of frequencies and percentages, while means and standard deviations were used to summarize continuous variables. The relative incidence (RI) of lymphadenopathy on the vaccination day (day 0) and during the risk period (days 1-42) was calculated using a conditional Poisson regression model. The potential influence of seasonality was controlled by including twelve 30-day calendar periods in the model. The combined effect of all three vaccine doses was also presented for each vaccine dose. Furthermore, we evaluated changes in the risk of lymphadenopathy over time from the vaccination date within the risk period (for days 1-7, 8-14, 15-28, and 29-42) by dose and combined effect.

Subgroup analyses were conducted to investigate the associations of baseline characteristics and vaccine types with the risk of lymphadenopathy on the vaccination day and during days 1-42. For the analysis by vaccine type, patients who received only one of the three types of vaccines—ChAdOx1, BNT162b2, or mRNA-1273—were included. In addition, the study evaluated changes in the RI with 7-day increases in the length of the risk period, ranging from 21 days to 63 days, for each dose and combined effect. All statistical analyses were performed using SAS Enterprise Guide 7.4 (SAS Institute Inc., Cary, NC, USA) and R version 4.1.3 (R Foundation for Statistical Computing, Vienna, Austria).

### Ethics statement

The study received ethical approval from the common institutional review board in Korea under the reference P01-202203-01-005, and written informed consent was waived for this study, because only de-identified data were used.

## RESULTS

### Characteristics of the study subjects

**Figure 1.**
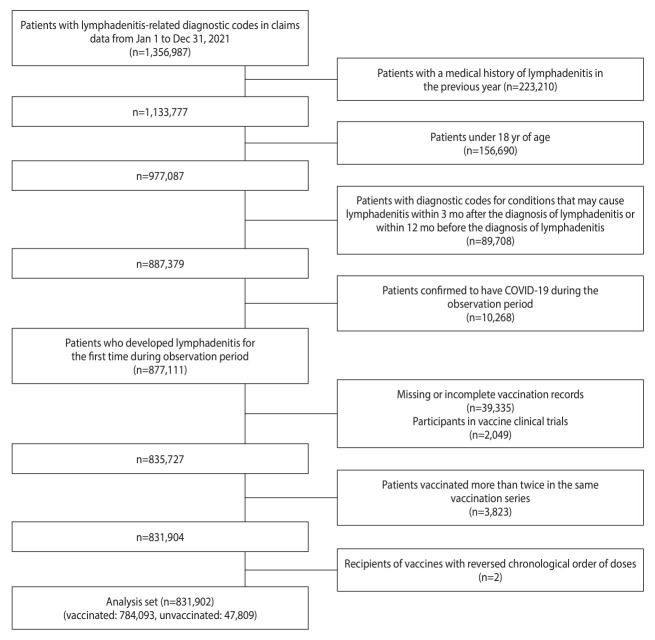
Flow chart of subject selection. COVID-19, coronavirus disease 2019.

Between January 2021 and December 2021, a substantial cohort of 1,356,987 patients received billing for lymphadenopathy-related diagnoses. Following exclusionary measures, 831,902 individuals were deemed eligible for analysis to explore the potential association between COVID-19 vaccination and lymphadenopathy. Among these subjects, 784,093 had been vaccinated against COVID-19, while 47,809 remained unvaccinated (Figure 1).

The characteristics of patient demographics are shown in Table 1. The results indicated a decline in the proportion of adverse events with advancing age, with 7.9% of cases reported in patients in their 70s and 4.3% in those aged 80 years or above. Female represented the majority of cases, accounting for 65.2%. The prevalence of hypertension, diabetes mellitus, and hyperlipidemia were 24.1%, 16.3%, and 33.8%, respectively. Most patients (95.9%) had a CCI, score of 0-4. In regards to vaccination, the most common regimen consisted of two doses of BNT162b2, followed by three doses of BNT162b2, two doses of mRNA-1273, and two doses of ChAdOx1 with a third dose of mRNA-1273, which accounted for 35.2%, 16.8%, 13.7%, and 10.1% of the patients, respectively. Fewer than 10% of cases had received one or fewer vaccinations, while 53.5% had received two vaccinations.

The length of the risk period and control period were 34.4±11.3 days and 286.0±26.2 days, respectively (Supplementary Material 2). The proportions of events were 24.3%, 74.8%, and 0.9% and the incidence rates per 1 person-year were 1.16 (95% CI, 1.16 to 1.17), 0.96 (95% CI, 0.95 to 0.96), and 1.46 (95% CI, 1.42 to 1.49) in the risk period, control periods, and the date of the vaccination, respectively (Table 1, Supplementary Material 2). Individuals with lymphadenopathy at the time of vaccination were found to have a higher proportion of being in the age range of 50 years to 60 years, male, and having received mRNA-1273 as their third dose following initial ChAdOx1 vaccination, in comparison to those who experienced lymphadenopathy during the risk or control periods. These observations were made through an analysis and comparison of data sets.

### The association between the coronavirus disease 2019 vaccine and the incidence of lymphadenopathy

The RI of lymphadenopathy increased with three successive vaccine doses. Among the administered doses, dose 3 demonstrated the highest RI (1.26; 95% CI, 1.24 to 1.28). The combined effect of vaccination on the incidence of lymphadenopathy was shown by an RI of 1.17 (95% CI, 1.17 to 1.18; Table 2) In addition, the relative risk of lymphadenopathy was observed to be higher on the day of vaccination than in the 1-42 days after vaccination with a combined effect of 1.47 (95% CI, 1.44 to 1.50). Stratifying the changing risk of lymphadenopathy over time from the day of vaccination, we observed an increasing risk with three successive doses only in the 1-day to 7-day period after vaccination (Table 3). Moreover, the risk of lymphadenopathy decreased with an increase in the time elapsed from the day of vaccination. The combined effect of the risk was estimated to be 1.59 (95% CI, 1.57 to 1.60), 1.18 (95% CI, 1.17 to 1.20), 1.04 (95% CI, 1.03 to 1.05), and 1.01 (95% CI, 1.00 to 1.02) for days 1-7, days 8-14, days 15-28, and days 29-42, respectively.

### Subgroup analysis and sensitivity analysis

The increased risk of lymphadenopathy with COVID-19 vaccination was statistically significant for age groups from the 20s through 60s, but no significant risk was observed in individuals over the age of 70. The increased risk was significant in both sexes, with females tending to present a greater risk. Moreover, the comorbidity index was found to be a significant factor, with an RI of 1.18 (95% CI, 1.17 to 1.19) for a comorbidity index of 0-4, and a non-significant RI of 1.02 (95% CI, 0.99 to 1.05) for a comorbidity index of 5 or more. An analysis of the combined effect of ChAdOx1 vaccines showed an RI of 1.01 (95% CI, 0.98 to 1.04), while the highest risk was associated with mRNA-1273 vaccines (RI, 1.43; 95% CI, 1.40 to 1.45) and BNT162b2 vaccines (RI, 1.19; 95% CI, 1.18 to 1.20; Table 4) A sensitivity analysis to assess the impact of the size of the risk window on the findings demonstrated a gradual decrease in the combined RI from 1.28 (95% CI, 1.28 to 1.29) to 1.14 (95% CI, 1.13 to 1.14) as the length of the risk window rose from 21 days to 63 days (Table 5).

## DISCUSSION

Our analysis revealed a statistically significant elevation in the risk of lymphadenopathy during the period of 1-42 days following vaccination. The risk was found to be significant only for the mRNA platform vaccines, such as BNT162b2 and mRNA-1273, and not for viral vector vaccines. The RI tended to be greater in younger and female individuals, as well as those receiving the third dose compared to the first dose. The highest risk was noted on the day of vaccination, although there was unclear temporal relationship between the vaccination and incidence. During the risk period, the RI for lymphadenopathy was highest in the first week, then declined gradually over time. The decreasing RI approached 1.00 by the end of the second week post-vaccination, indicating a negligible magnitude of lymphadenopathy risk in spite of the statistical significance.

As of October 31, 2021, a total of 39,739,672 individuals had received COVID-19 vaccines in Korea. In this study, 209,833 cases with lymphadenopathy within 42 days after vaccination were identified, accounting for 0.5%. While this proportion is considerably lower than the reported incidence of radiologically detectable lymphadenopathy (9-54%) [20], it aligns with a study that reported the incidence of clinically detectable lymphadenopathy to be 0.4% [21].

The characteristics of lymphadenopathy observed in our analysis are in line with studies that have investigated lymphadenopathy following COVID-19 vaccination. A prospective evaluation of MRI scans before and after administration of the COVID-19 vaccine revealed that being female, and having a younger age were identified as predisposing factors for vaccination-related axillary lymphadenopathy in individuals who received two doses of either the BNT162b2 or mRNA-1273 vaccine [19]. Lymphadenopathy risk decreased gradually at 2 weeks post-vaccination [19]. A survey performed in Vietnam found that recipients of the third dose of COVID-19 vaccines had a significantly higher odds ratio of experiencing lymphadenopathy within 14 days of vaccination than those who received the first and second doses [22]. A meta-analysis of studies reporting adverse events following COVID-19 vaccination in Saudi Arabia demonstrated that the risk of lymphadenopathy was significantly higher with BNT162b2 than with ChAdOx1 [23].

Previous studies evaluating the causal relationship between the administration of mRNA-based COVID-19 vaccines and lymphadenopathy also reported similar results to our study. In a phase 3 clinical trial conducted in the United States involving 30,351 individuals who received the mRNA-1273 vaccine or a placebo, axillary swelling or tenderness was evaluated within 7 days of vaccination. The incidence was notably higher in the mRNA-1273 vaccinated group, with rates of 10.2% and 14.2% observed after the first and second doses compared to the control group, with rates of 4.8% and 3.9% after the first and second doses, respectively. The mean time to outcome for the vaccinated group was 2.3 days after the first dose and 2.4 days after the second dose [11]. Furthermore, a targeted trial emulation analysis of 823,006 individuals vaccinated with BNT162b2 and 823,006 unvaccinated individuals in Israel demonstrated a significantly higher risk of lymphadenopathy within 42 days of vaccination in the vaccinated group (2.43; 95% CI, 2.05 to 2.78) [10]. Finally, a selfcontrolled risk interval analysis of electronic health record data from 47,999 individuals who received three doses of mRNA vaccines in the United States revealed a significantly increased risk of lymphadenopathy, with a higher proportion of individuals experiencing this condition after the third dose compared to the second dose [12].

The biological mechanism of COVID-19 vaccine-associated lymphadenopathy has not been conclusively established, but it is primarily considered to be attributed to reactive hyperplasia, characterized by the rapid expansion of T and B cells. The mRNA vaccine exhibited greater potency in generating antigen-specific antibodies and T cell responses than the adenovirus vector vaccine [20]. This supports the disparity in the risk of lymphadenopathy between vaccine platforms.

The present study offered notable advantages in terms of high internal validity by employing statistical analysis techniques to adjust for all time-invariant confounders. The external validity of this study is also strengthened by including all cases of lymphadenopathy that occurred during the study period. However, the findings of this study must be interpreted considering the following limitations. Firstly, with respect to the increased risk observed on the day of vaccination, data to determine whether vaccination or lymphadenopathy occurred first were not available from the NHIS database. We found a trend toward increased risk closer to the timing of vaccination, but the risk on the day of vaccination should be interpreted with caution. Secondly, the immune response triggered by vaccine components might have resulted in swelling of the axillary LNs, which are nearest to the usual vaccination site. However, since the specific location of the lymphadenopathy (i.e., cervical, inguinal, or axillary) was not available in the data, there was a potential for overestimating the causal association by including lymphadenopathy as an outcome within the risk period. Thirdly, all cases of lymphadenopathy, regardless of severity, for which patients visited healthcare providers were included in the study’s outcome. Therefore, it remained uncertain whether mild cases of lymphadenopathy within the range of the normal immune response should be classified as adverse events. In relation to severe cases, the diagnostic, therapeutic, and prognostic processes might be of clinical interest, but were not addressed in this study, highlighting the need for further investigation.

In conclusion, there has been a significant increase in the incidence of lymphadenopathy associated with COVID-19 vaccination. Moreover, research has shown that the risk of lymphadenopathy is significant only for mRNA platform vaccines, not for viral vector vaccines, and it is higher among younger people and females. These findings suggest a possible epidemiologic causal relationship, while they also challenge healthcare providers to differentiate between lymphadenopathy caused by an immune response to mRNA vaccines and that caused by a pathologic process.

## Figures and Tables

**Table 1.** Characteristics of patient demographics in relation to the incidence of lymphadenopathy during the risk and control periods after coronavirus disease 2019 vaccination

Characteristics	Total	Events on day 0	Events in risk period	Events in control period
Total (n)	831,902	7,417	202,466	622,019
Age (yr)				
Mean±SD	46.9±17.9	47.7±16.6	47.3±17.6	46.7±18.0
18-29	178,417 (21.5)	1,397 (18.8)	41,009 (20.3)	136,011 (21.9)
30-39	142,069 (17.1)	1,140 (15.4)	33,546 (16.6)	107,383 (17.3)
40-49	151,429 (18.2)	1,344 (18.1)	38,079 (18.8)	112,006 (18.0)
50-59	140,421 (16.9)	1,549 (20.9)	35,668 (17.6)	103,204 (16.6)
60-69	118,207 (14.2)	1,306 (17.6)	29,856 (14.8)	87,045 (14.0)
70-79	65,641 (7.9)	476 (6.4)	16,288 (8.0)	48,877 (7.9)
≥80	35,718 (4.3)	205 (2.8)	8,020 (4.0)	27,493 (4.4)
Sex				
Male	289,192 (34.8)	2,802 (37.8)	67,813 (33.5)	218,577 (35.1)
Female	542,710 (65.2)	4,615 (62.2)	134,653 (66.5)	403,442 (64.9)
Comorbidities				
Hypertension	200,650 (24.1)	1,890 (25.5)	49,238 (24.3)	149,522 (24.0)
Diabetes mellitus	135,691 (16.3)	1,225 (16.5)	33,038 (16.3)	101,428 (16.3)
Hyperlipidemia	280,907 (33.8)	2,571 (34.7)	69,941 (34.5)	208,395 (33.5)
Charlson comorbidity index				
Mean±SD	1.0±1.5	1.0±1.5	1.0±1.5	1.0±1.5
0-4	797,495 (95.9)	7,138 (96.2)	194,346 (96.0)	596,011 (95.8)
≥5	34,407 (4.1)	279 (3.8)	8,120 (4.0)	26,008 (4.2)
Type of vaccination				
ChAd	1,317 (0.2)	4 (0.1)	301 (0.2)	1,012 (0.2)
ChAd/ChAd	24,550 (3.0)	194 (2.6)	5,690 (2.8)	18,666 (3.0)
ChAd/ChAd/ChAd	4 (0.0)	1 (0.0)	(0.0)	3 (0.0)
ChAd/ChAd/mRNA-1273	84,003 (10.1)	978 (13.2)	23,133 (11.4)	59,892 (9.6)
ChAd/ChAd/BNT	47,881 (5.8)	502 (6.8)	14,207 (7.0)	33,172 (5.3)
ChAd/mRNA-1273	3 (0.0)	-	2 (0.0)	1 (0.0)
ChAd/mRNA-1273/mRNA-1273	4 (0.0)	-	-	4 (0.0)
ChAd/BNT	12,038 (1.5)	97 (1.3)	3,169 (1.6)	8,772 (1.4)
ChAd/BNT/mRNA-1273	191 (0.0)	1 (0.0)	54 (0.0)	136 (0.0)
ChAd/BNT/BNT	25,677 (3.1)	282 (3.8)	7,934 (3.9)	17,461 (2.8)
Ad26	2,428 (0.3)	15 (0.2)	320 (0.2)	2,093 (0.3)
Ad26/BNT	2 (0.0)	-	-	2 (0.0)
mRNA-1273	3,607 (0.4)	10 (0.1)	951 (0.5)	2,646 (0.4)
mRNA-1273/ChAd	1 (0.0)	-	-	1 (0.0)
mRNA-1273/mRNA-1273	113,777 (13.7)	1,084 (14.6)	31,282 (15.5)	81,411 (13.1)
mRNA-1273/mRNA-1273/mRNA-1273	17,181 (2.1)	206 (2.8)	4,887 (2.4)	12,088 (1.9)
mRNA-1273/mRNA-1273/BNT	1,266 (0.2)	7 (0.1)	410 (0.2)	849 (0.1)
mRNA-1273/BNT	2,142 (0.3)	23 (0.3)	537 (0.3)	1,582 (0.3)
BNT	15,368 (1.9)	82 (1.1)	1,818 (0.9)	13,468 (2.2)
BNT/ChAd	3 (0.0)	-	1 (0.0)	2 (0.0)
BNT/ChAd/BNT	1 (0.0)	-	-	1 (0.0)
BNT/Ad26	1 (0.0)	-	1 (0.0)	-
BNT/mRNA-1273	2 (0.0)	-	-	2 (0.0)
BNT/BNT	292,703 (35.2)	2,623 (35.4)	70,483 (34.8)	219,597 (35.3)
BNT/BNT/mRNA-1273	560 (0.1)	4 (0.1)	134 (0.1)	422 (0.2)
BNT/BNT/BNT	139,382 (16.8)	1,303 (17.6)	37,152 (18.4)	100,927 (16.2)
Vaccine doses				
No vaccination	47,809 (5.8)	-	-	47,809 (7.7)
1st dose only	22,720 (2.7)	111 (1.5)	3,390 (1.7)	19,219 (3.1)
2nd dose	445,222 (53.5)	4,021 (54.2)	111,165 (54.9)	330,036 (53.1)
3rd dose	316,151 (38.0)	3,285 (44.3)	87,911 (43.4)	224,955 (36.2)

Values are presented as number (%). ChAd, ChAdOx1; BNT, BNT162b2; Ad26, Ad26.COV2.S; SD, standard deviation.

**Table 2.** Risk of lymphadenopathy by coronavirus disease 2019 vaccination

Period	Dose 1	Dose 2	Dose 3	Combined effect
Control period	1.00 (reference)	1.00 (reference)	1.00 (reference)	1.00 (reference)
Day 0	1.35 (1.30, 1.40)	1.53 (1.48, 1.59)	1.60 (1.51, 1.68)	1.47 (1.44, 1.50)
Day 1 to 42	1.19 (1.18, 1.20)	1.15 (1.14, 1.16)	1.26 (1.24, 1.28)	1.17 (1.17, 1.18)

Values are presented as relative incidence (95% confidence interval).

**Table 3.** The risk of developing lymphadenopathy after coronavirus disease 2019 vaccination by time since vaccination

Risk-period (day)	Dose 1	Dose 2	Dose 3	Combined effect
1 to 7	1.37 (1.35, 1.39)	1.77 (1.74, 1.79)	1.72 (1.68, 1.75)	1.59 (1.57, 1.60)
8 to 14	1.42 (1.40, 1.44)	1.02 (1.00, 1.04)	0.94 (0.91, 0.97)	1.18 (1.17, 1.20)
15 to 28	1.07 (1.06, 1.09)	1.03 (1.02, 1.04)	0.95 (0.92, 0.98)	1.04 (1.03, 1.05)
29 to 42	1.03 (1.01, 1.05)	1.00 (0.99, 1.02)	0.89 (0.83, 0.94)	1.01 (1.00, 1.02)

Values are presented as relative incidence (95% confidence interval).

**Table 4.** Subgroup analysis on the risk of lymphadenopathy after coronavirus disease 2019 vaccination

Subgroup	Dose 1		Dose 2		Dose 3		Combined dose	
	Day 0	Day 1-42	Day 0	Day 1-42	Day 0	Day 1-42	Day 0	Day 1-42
Age (yr)								
18-29	1.32 (1.22, 1.43)	1.25 (1.22, 1.27)	1.48 (1.37, 1.60)	1.16 (1.14, 1.18)	1.58 (1.33, 1.87)	2.19 (2.09, 2.29)	1.42 (1.34, 1.49)	1.23 (1.21, 1.25)
30-39	1.53 (1.40, 1.67)	1.36 (1.33, 1.38)	1.69 (1.55, 1.84)	1.28 (1.26, 1.30)	1.83 (1.50, 2.24)	1.91 (1.81, 2.03)	1.62 (1.53, 1.72)	1.33 (1.31, 1.35)
40-49	1.53 (1.41, 1.66)	1.22 (1.20, 1.25)	1.72 (1.59, 1.86)	1.27 (1.25, 1.30)	1.31 (1.08, 1.57)	2.01 (1.91, 2.10)	1.60 (1.52, 1.69)	1.28 (1.26, 1.30)
50-59	1.47 (1.35, 1.59)	1.13 (1.11, 1.15)	1.92 (1.77, 2.07)	1.14 (1.12, 1.16)	1.86 (1.66, 2.09)	1.59 (1.53, 1.66)	1.73 (1.65, 1.82)	1.18 (1.16, 1.20)
60-69	1.23 (1.11, 1.36)	0.99 (0.97, 1.02)	1.53 (1.40, 1.68)	1.02 (0.99, 1.04)	1.73 (1.57, 1.90)	1.03 (1.00, 1.07)	1.48 (1.40, 1.56)	1.01 (1.00, 1.03)
70-79	0.70 (0.58, 0.83)	0.97 (0.94, 1.00)	0.78 (0.66, 0.92)	0.98 (0.95, 1.00)	1.37 (1.19, 1.57)	0.96 (0.92, 1.00)	0.92 (0.84, 1.01)	0.97 (0.95, 0.99)
≥80	0.45 (0.33, 0.60)	0.92 (0.87, 0.96)	0.54 (0.41, 0.70)	0.96 (0.92, 1.00)	1.53 (1.26, 1.85)	0.96 (0.91, 1.01)	0.77 (0.67, 0.88)	0.95 (0.92, 0.98)
Sex								
Male	1.48 (1.40, 1.58)	1.11 (1.10, 1.13)	1.65 (1.56, 1.75)	1.14 (1.13, 1.16)	1.74 (1.60, 1.89)	1.18 (1.15, 1.22)	1.60 (1.54, 1.66)	1.13 (1.12, 1.15)
Female	1.28 (1.22, 1.34)	1.23 (1.21, 1.24)	1.47 (1.41, 1.54)	1.15 (1.14, 1.16)	1.52 (1.42, 1.62)	1.30 (1.28, 1.33)	1.40 (1.36, 1.44)	1.19 (1.19, 1.20)
Comorbidities								
Hypertension	1.16 (1.07, 1.25)	1.06 (1.04, 1.08)	1.29 (1.19, 1.39)	1.06 (1.04, 1.08)	1.63 (1.50, 1.77)	0.98 (0.95, 1.01)	1.33 (1.27, 1.39)	1.05 (1.04, 1.06)
Diabetes mellitus	1.16 (1.05, 1.28)	1.06 (1.04, 1.08)	1.26 (1.15, 1.38)	1.07 (1.05, 1.09)	1.53 (1.38, 1.70)	0.96 (0.93, 0.99)	1.29 (1.22, 1.37)	1.05 (1.03, 1.06)
Dyslipidemia	1.19 (1.11, 1.27)	1.11 (1.09, 1.12)	1.34 (1.26, 1.43)	1.09 (1.07, 1.10)	1.59 (1.47, 1.71)	1.04 (1.01, 1.06)	1.34 (1.29, 1.39)	1.09 (1.08, 1.10)
Charlson comorbidity index								
0-4	1.37 (1.32, 1.42)	1.19 (1.18, 1.20)	1.55 (1.50, 1.61)	1.15 (1.14, 1.16)	1.60 (1.51, 1.69)	1.30 (1.28, 1.32)	1.48 (1.45, 1.52)	1.18 (1.17, 1.19)
≥5	0.96 (0.77, 1.18)	1.03 (0.99, 1.08)	1.04 (0.85, 1.28)	1.04 (1.00, 1.08)	1.62 (1.33, 1.97)	0.96 (0.90, 1.03)	1.16 (1.03, 1.31)	1.02 (0.99, 1.05)
Type of vaccination								
ChAdOx1	1.41 (1.16, 1.70)	1.01 (0.97, 1.05)	1.36 (1.11, 1.67)	1.01 (0.97, 1.05)	N/A	N/A	1.39 (1.21, 1.60)	1.01 (0.98, 1.04)
mRNA-1273	1.54 (1.42, 1.68)	1.59 (1.56, 1.62)	2.11 (1.95, 2.27)	1.28 (1.25, 1.30)	1.55 (1.21, 1.98)	1.78 (1.64, 1.94)	1.78 (1.69, 1.89)	1.43 (1.40, 1.45)
BNT162b2	1.36 (1.29, 1.42)	1.14 (1.12, 1.15)	1.5 (1.43, 1.57)	1.19 (1.18, 1.20)	1.58 (1.46, 1.72)	1.41 (1.38, 1.45)	1.45 (1.41, 1.50)	1.19 (1.18, 1.20)

Values are presented as relative incidence (95% confidence interval). N/A, not available.

**Table 5.** Sensitivity analysis of lymphadenopathy risk by the length of the risk window

Risk window	RI (95% CI) for day 1-42			
	Dose 1	Dose 2	Dose 3	Combined effect
Days 21	1.29 (1.28, 1.30)	1.27 (1.26, 1.28)	1.31 (1.29, 1.33)	1.28 (1.28, 1.29)
Days 28	1.23 (1.22, 1.25)	1.21 (1.20, 1.22)	1.28 (1.26, 1.31)	1.23 (1.22, 1.24)
Days 35	1.20 (1.20, 1.21)	1.17 (1.17, 1.18)	1.27 (1.25, 1.29)	1.20 (1.19, 1.20)
Days 42	1.19 (1.18, 1.20)	1.15 (1.14, 1.16)	1.26 (1.24, 1.28)	1.17 (1.17, 1.18)
Days 49	1.18 (1.17, 1.19)	1.13 (1.12, 1.14)	1.26 (1.24, 1.28)	1.16 (1.15, 1.17)
Days 56	1.17 (1.16, 1.18)	1.11 (1.11, 1.12)	1.27 (1.25, 1.29)	1.15 (1.14, 1.15)
Days 63	1.16 (1.15, 1.17)	1.10 (1.10, 1.11)	1.27 (1.25, 1.29)	1.14 (1.13, 1.14)

RI, relative incidence; CI, confidence interval.

## SUPPLEMENTARY MATERIALS

Supplement Material 1. List of ICD-10 codes for variables

Supplement Material 2. Follow-up duration, number of events, and incidence rate (per 1 person-year) of lymphadenopathy by period
