# Supplementary material for: Risk of lymphadenopathy from SARS-CoV-2 vaccination in Korea: a self-controlled case series analysis
Source: Epidemiol Health. 2023 Oct 13;45:e2023090. doi: 10.4178/epih.e2023090 (PMC10867511; doi:10.4178/epih.e2023090)
Supplement: Supplement Material 1. — List of ICD-10 codes for variables [file epih-45-e2023090-Supplementary-1.docx]

Supplementary Material 1. List of ICD-10 codes for variables

| Comorbidities | ICD-10 code |
| --- | --- |
| Major comorbidities |  |
| Diabetes mellitus | E10, E11, E13, E14 |
| Dyslipidemia | E78.0, E78.1, E78.2, E78.3, E78.4, E78.5 |
| Hypertension | I10, I11, I12, I13, I15 |
| Exclusion criteria |  |
| Tuberculosis | A15.4, A16.3, A18.2, A18.3, K93.0 |
| Plague | A20.0 |
| Tularemia | A21.0, A21.1 |
| Chlamydial infection | A55 |
| HIV disease | B21.1, B21.2, B21.3, B23.1 |
| Sporotrichosis | B42.1 |
| Malignant neoplasm | C00-C75, C46.3, C77, C81-C96 |
| Hemangioma and lymphangioma | D18 |
| Other neoplasms of lymphoid, hematopoietic tissue | D47 |
| Sarcoidosis | D86.1, D86.2 |
| Postmastectomy lymphoedema syndrome | I97.2 |
