# Supplementary material for: Risk of lymphadenopathy from SARS-CoV-2 vaccination in Korea: a self-controlled case series analysis
Source: Epidemiol Health. 2023 Oct 13;45:e2023090. doi: 10.4178/epih.e2023090 (PMC10867511; doi:10.4178/epih.e2023090)
Supplement: Supplement Material 2. — Follow-up duration, number of events, and incidence rate (per 1 person-year) of lymphadenopathy by period [file epih-45-e2023090-Supplementary-2.docx]

Supplementary Material 2. Follow-up duration, number of events, and incidence rate (per 1 person-year) of lymphadenopathy by period

|  | Control period | Risk period | | | | | | | |
| --- | --- | --- | --- | --- | --- | --- | --- | --- | --- |
|  |  | Dose 1 | | Dose 2 | | Dose 3 | | Combined effect | |
|  |  | Day 0 | Day 1-42 | Day 0 | Day 1-42 | Day 0 | Day 1-42 | Day 0 | Day 1-42 |
| Follow-up duration,  mean (SD) | 286.0 (26.2) | 1 (0) | 34.4 (8.4) | 1 (0) | 41.5 (3.8) | 1 (0) | 16.6 (10.9) | 1 (0) | 34.4 (11.3) |
| Number of events | 622019 | 2840 | 86144 | 3185 | 98918 | 1392 | 17404 | 7417 | 202466 |
| Incidence rate/PY  (95% CI) | 0.96  (0.95-0.96) | 1.32  (1.28-1.37) | 1.17  (1.16-1.17) | 1.53  (1.48-1.58) | 1.15  (1.14-1.15) | 1.61  (1.53-1.69) | 1.27  (1.25-1.28) | 1.46  (1.42-1.49) | 1.16  (1.16-1.17) |
